# Editorial: Ageing and migration status: Intersectional forms of discrimination and exclusion

**DOI:** 10.3389/fpsyg.2023.1150578

**Published:** 2023-02-21

**Authors:** Matt Flynn, Petia Genkova, Elaine Dewhurst, Christoph Daniel Schaefer

**Affiliations:** ^1^Centre for Research into the Older Workforce, University of Hull, Hull, United Kingdom; ^2^Faculty of Business Management and Social Sciences, Osnabrück University of Applied Sciences, Osnabrück, Germany; ^3^Faculty of Humanities, The University of Manchester, Manchester, United Kingdom

**Keywords:** active ageing, ageing in place, migration, empowerment, inclusion, older migrants, barriers for older migrants, intersectionality

Populations of many countries around the world are faced with ageing populations. The proportion of people aged over 60 years in 2050 is projected to be nearly twice as high as it was in 2012 (namely 22% vs. 12%; World Health Organization, 2022). In economically developed countries, these proportions are even higher. According to a projection, the share of people aged 60 or older is estimated to rise from 26.0% in 2021 to 34.3% in 2050 (United Nations, Department of Economic and Social Affairs, 2022). As populations age, societies are confronted with challenges in terms of social insurance systems, health, and societal cohesion. These demographic trends and the entailed challenges are leading to a growing interest amongst policymakers, businesses, and other stakeholders on ways to enable older people to “actively age” through: interventions to promote healthy ageing; participation in social, economic, and civic affairs; and ensuring physical, social and income security. Older people's access to resources necessary for ageing well is impacted by socio-economic status. This in turn, draws attention to the policy and resource needs of communities of older people. Communities which are particularly vulnerable to isolation and a shortage of recourses are older immigrants. These communities include economic migrants, asylum seekers, and undocumented workers. For many, their life courses are characterized by precarious and disrupted careers, inaccessibility to public resources, and social isolation, in addition to age and race intersecting to create unique forms of discrimination.

This Research Topic explores the barriers which elderly people face to ageing well and potential public and social policies for ensuring safe, participative, and healthy ageing. It thus contributes to the dialogue on the ways in which policy makers, businesses, third sector organizations and elderly people themselves can enhance active ageing. The articles constituting this Research Topic focus in particular on barriers to the wellbeing and social inclusion of elderly people, and on potential measures for supporting healthy and active ageing.

Addressing elderly people's wellbeing in relation to health, Chen et al. examines the relationship between self-efficacy, sports participation, and health promotion behavior for the middle-aged and elderly. The author found that the perceived self-efficacy of middle-aged and elderly people positively affected health promotion behavior. This relationship between self-efficacy and health promotion behavior was partially mediated by participation in sports. Chen et al. suggests to promoting sports participation of elderly people to improve public health.

Also addressing aspects related to health, Zhao et al. study the associations between social support, psychological capital and self-neglect. Their research coincides with the previous article insofar as psychological capital was partially operationalised as self-efficacy. The authors found that social support and psychological capital can mitigate against self-neglect, with psychological capital functioning as a mediator between social support and self-neglect. They suggest that social support and higher psychological capital could decrease the risk of self-neglect in older adults.

Focusing on the interrelationship between social factors and psychological wellbeing, Wang et al. explore how social exclusion, the sense of belonging, and suicidal ideation are associated with one another. They found that social exclusion increased the suicidal ideation of elderly. Both the sense of belonging and depression acted as mediators in the relationship between social exclusion and suicidal ideation. Additionally, the authors found that interpersonal trust acts as a buffer against the effects of social exclusion on the sense of belonging, depression, and suicidal ideation.

Cheng and Zhang examine the effects of finance on wellbeing, namely the effect of a pension insurance, more than 10 years after its introduction. They found that the effect of participation in the pension insurance on subjective wellbeing depended on framework conditions. The participants who profited most from the pension insurance were those with a poor health status, but otherwise good overall conditions. The authors therefore recommend implementing social security policies according to local conditions.

Aspects which are also related to the socioeconomic situation of elderly are examined by Flynn and Wong. The authors explore how a community organization can foster the social and economic participation of elderly. In particular, they studied how a community organization can overcome employment barriers of older immigrants. Older immigrants often face multiple barriers to work due to age and migration. The authors found that a community organization identified these barriers toward employability and reduce these barriers by offering elderly immigrants skill training and qualifications.

A more general obstacle toward the social and economic inclusion of elderly is formed by stereotypes about elderly. Shimizu indicates that the elderly are often associated with negative traits, such as incompetence and stubbornness. The author points out that people differ in how long off they perceive the period until they become elderly, even when they are of the same age. The author suggests that this subjective distance could affect stereotypes against elderly and therefore proposes to focus on this subjective distance to find more effective intervention methods.

Alises et al. analyse how first- and second-generation immigrants differ in terms of the assimilation of their behavior. They found that first-generation immigrants in Portugal evince a higher degree of delinquency than the Portuguese majority population, but that there is no difference between second-generation immigrants and the majority population. These results can suggest that social circumstances matter more in terms of delinquent behavior than individual factors.

Further aspects that centrally affect the inclusion of immigrants relate to the characteristics of the majority population. Genkova and Schreiber examine the attitudes and competences of employees toward diversity. They found that employees in the STEM sector have poor competences in dealing with diversity in their daily work. This seems to be associated with a lack of experience and a lack of support from the respective organizations/leaders. The authors suggest that diversity competences should be strengthened, to be able to utilize the potential of heterogeneous working groups.

Johansson et al. examine a specific aspect of diversity competence, namely cultural sensitivity, emphasizing the role of cultural sensitivity in assisting in active ageing. They found that municipal officials in Sweden preferred not to use cultural sensitivity as a concept in their work, instead tailoring interventions based on individual preferences. The authors suggest that emphasis drifts away from personal preferences toward knowledge about cultures.

A theme connecting several of these contributions is the challenge which immigrant communities have in drawing on resources to age in a healthy way. The papers in this Research Topic point to measures which government, employers, community groups, second generation family members and immigrants can take to both foster healthy ageing and promote integration and social cohesion. Interventions identified in these papers include: intergenerational support; intercultural solidarity; assimilation; community activism; community involvement; cultural sensitivity; diversity competences; self-efficacy and government outreach to migrant communities. Through a patchwork of help from these different stakeholders, older immigrants can access the resources both within and outside of their communities so that they can have lives which they value.

We hope this Research Topic has cast a light on the lived experiences of a community of older people who are often overlooked in terms of public and social policy discussions on ageing. Further research is needed to better understand both how different immigrant groups age and how stakeholders can better support them. We would point to two areas where knowledge can be further advanced: first, several of the papers we showcased discuss the experiences of “aged in place” immigrants—namely those who have faced lifetimes of disruption, precarity, and exclusion. These are the experiences of many, but not necessarily all immigrant groups. Some more recent cohorts of immigrants (for example BNO Hong Kong immigrants to the United Kingdom) are receiving government and third-party help in the forms of training and accreditation, language help, housing and cultural assimilation. Others migrate as professionals with skills which employers need and strive to maintain. It is worth exploring how those interventions overcome barriers which have been identified in these papers. Second, the papers also show how older immigrants can both individually and collectively co-produce healthy ageing lives which they value and how, with resources from stakeholders, they can have voices in their wider communities. We therefore think that there is a case for further research on the tools which older immigrants need in order to have agency and voice over their lives and to address the barriers which many have faced to involvement in their host communities.

## Author contributions

All authors listed have made a substantial, direct, and intellectual contribution to the work and approved it for publication.
